# Microfluidics and Nanomaterial-based Technologies for Circulating Tumor Cell Isolation and Detection

**DOI:** 10.3390/s20071875

**Published:** 2020-03-27

**Authors:** Sheng-Jen Cheng, Kuan Yu Hsieh, Shiue-Luen Chen, Chong-You Chen, Chien-Yu Huang, Hung-I Tsou, Priyank V. Kumar, Jason Chia-Hsun Hsieh, Guan-Yu Chen

**Affiliations:** 1 Department of Electrical and Computer Engineering, College of Electrical and Computer Engineering, National Chiao Tung University, Hsinchu 30010, Taiwan; shengjen@nctu.edu.tw (S.-J.C.); Kuan.Yu@ibm.com (K.Y.H.); g199500@gmail.com (S.-L.C.); ericchen161206@gmail.com (C.-Y.C.); apple21038526@gmail.com (C.-Y.H.); a097683164924@gmail.com (H.-I.T.); 2 Institute of Biomedical Engineering, College of Electrical and Computer Engineering, National Chiao Tung University, Hsinchu 30010, Taiwan; 3 School of Chemical Engineering, University of New South Wales, Sydney, NSW 2052, Australia; priyank.kumar@unsw.edu.au; 4 Division of Haematology/Oncology, Department of Internal Medicine, Chang Gung Memorial Hospital (Linkou), Taoyuan 333, Taiwan; 5 Department of Biological Science and Technology, National Chiao Tung University, Hsinchu 30010, Taiwan

**Keywords:** liquid biopsy, circulating tumor cells, microfluidic, graphene oxide, surface modification

## Abstract

Cancer has been one of the leading causes of death globally, with metastases and recurrences contributing to this result. The detection of circulating tumor cells (CTCs), which have been implicated as a major population of cells that is responsible for seeding and migration of tumor sites, could contribute to early detection of metastasis and recurrences, consequently increasing the chances of cure. This review article focuses on the current progress in microfluidics technology in CTCs diagnostics, extending to the use of nanomaterials and surface modification techniques for diagnostic applications, with an emphasis on the importance of integrating microchannels, nanomaterials, and surface modification techniques in the isolating and detecting of CTCs.

## 1. Introduction

Cancer is a complex disease which involves abnormal growth of cells through a multi-step evolutionary process. It has been characterized as a heterogeneous disease consisting of many different subtypes with the potential to invade or spread to distant parts of the body. Almost all cancers can metastasize, and it has been estimated that 80% of cancer-related deaths are due to metastases [1]. The metastatic ability make cancer a leading cause of death worldwide. Globally, in 2012, there were 14.1 million new cancer cases and 8.2 million cancer-related deaths. The World Health Organization estimated that, by 2035, the world could see 24 million new cancer cases and 14.5 million cancer-related deaths a year [2,3].

The early detection of patients with breast, colorectal, gastric, and liver cancer, has a better prognosis and increased life expectancy [4]. Recently, liquid biopsy, including CTCs, cell-free DNA (cfDNA) and extracellular vehicles (EVs), has attracted more attention due to its minimal-invasiveness and bio-informatics affluence [5]. Compared with traditional ‘solid biopsy’, which cannot always be performed to determine tumor dynamics, liquid biopsy has notable advantages in its noninvasive modality [6,7,8]. Moreover, studies have found the changes in CTC numbers before and after chemotherapy and during follow-up might predict cancer relapse [9,10]. Ried et al. reported that CTCs were detected in patients with high risk of developing cancer. In this case, different type of cancer patients with a useful screening tool for the early detection of cancer in patients with a higher risk profile [11]. These suggest that CTCs are one of the important indicators for early detection, monitoring cancer progression and evaluating treatment effectiveness.

Despite CTCs’ promising clinical application, their rarity makes the detection extremely challenging. In 7.5 mL of blood from cancer patient, probably only 1 to 10 CTCs exisits [12], along with billions of red blood cells (RBCs) and millions of white blood cells (WBCs). Since the discovery of CTCs in 1869 [13], a large number of techniques have been developed to analysis them. Most of the strategies include the isolation of targets from body fluids and followed by characterization and analysis of the isolated targeted cells.

Here, we elucidate the physical and biological properties of CTCs. We review different microfluidic-based technologies for CTCs isolation and detection. Besides, we put emphasis on how integrating nanobiological interface to microfluidic systems could improve specificity and sensitivity in CTCs enumeration and analysis. We also discuss the importance of a standardized approach in promoting the transition of basic bench-side technologies into clinical applications.

## 2. Circulating Tumor Cells (CTCs)

### 2.1. Formation of CTCs

Cancer metastasis is a multi-step, stochastic process through which invasive tumor cells detach from the primary tumor and enter the circulation to invade distant tissue sites. Two biological mechanisms are involved in releasing CTCs from tumor site into blood stream, they are epithelial-mesenchymal transition (EMT) and non-EMT mediated invasion. The metastatic potential of tumor cells may be entirely shaped at the primary tumor site, or also by signaling events occurring during the intravascular transit of tumor cells. Tumor cells process local invasion, followed by intravasation to enter the circulatory system [14]. When cancerous cells migrate in the circulation, they may interact with platelets, lymphocytes, and other blood components, and eventually reach their distant organs and begin proliferation. Though the process of metastasis has been more familiarized, the mechanism governing organ-specific metastasis is still poorly understood [15,16].

### 2.2. Biological Properties of CTCs-Heterogeneity of CTCs

CTCs are rare cell species present in peripheral blood and appear in circulatory system during cancer metastasis. CTCs are cancer cells discovered in cancer patients’ peripheral blood that successfully escape from the primary tumor site and lead to metastases, these CTCs struggle to survive in the bloodstream, and have potential for seeding metastases [17,18]. They must survive a variety of, epithelial cell normally adhere to the extracellular matrix via integrin and this is essential for cell survival. In the absence of such an anchor, epithelial cells undergo anoikis, a form of cell death triggered by loss of anchorage to the substrate. Therefore, CTCs must develop a mechanism of resistance against anoikis. Once into the parenchyma of distant organs, CTCs can initiate the growth of a secondary tumor. However, in most cases the dispersed tumor cells enter a state of dormancy in the form of micro-metastases. These cells are resistant to traditional chemotherapy that targets rapidly dividing cells and are responsible for the development of late metastases [19].

CTCs profile changes during the above-mentioned processes. During EMT, tumor cells have a reduced expression of epithelial markers to initiate the dissemination from their primary sites [20]. Disseminated cells may partially revert to the epithelial phenotype through mesenchymal-epithelial transition (MET) to facilitate the proliferation after arriving the secondary sites [21]. Cancer cells during these transitions might show partial EMT phenotype, with both epithelial and mesenchymal features [22]. Vimentin (a member of the intermediate filament family) [23] and N-cadherin (a transmembrane protein) [24] are both expressed in mesenchymal cells. Several EMT-initaiting transcription factors (TF), such as Twist, Slug, Zeb1, Snail1 and FoxC2 are also found overexpressed in CTCs [25].

In addition to the most commonly found CTCs, researchers have recently focused on studying circulating tumor microemboli (CTM), which are metastatic CTCs in the form of clusters of cells. Despite the formation of CTMs and their contribution to metastasis are not fully defined yet, clinical studies have shown the presence of CTMs are associated with worse prognosis in patients [26,27]. Using mouse models, researchers demonstrated that CTM arise from oligoclonal tumor cell groupings and have 23- to 50-fold increased metastatic potential when compared with single CTCs. [28]. In addition, CTMs could not only pass through capillary-sized vessels but also rapidly recover after infiltration from a compressed form to a cluster of agglomerated cells, invading the surrounding tissue as they grow [29]. They are highly resistant to blood flow shear stress owing to their strong cell-to-cell adhesion and anti-anoikis capacities [30], allowing them to imitate normal cells to avoid immune surveillance. In addition, CTMs and CTCs respond differently to chemotherapy agents. CTMs have a higher survival rate than CTCs after drug treatment, indicating that they have some degree of drug resistance [31], and a survival advantage over most CTCs while circulating in blood stream [32].

As a means of liquid biopsy, CTCs could play a major role in the development of personalized medicine and targeted therapies, molecular comparison of CTCs to individual cells in CTMs might provide new insights into cancer biology [33]. With the advent of new sensitive technologies that permit live cell isolation and downstream genomic analysis, the existing CTCs related researches had evolved to explore clinical utility of these cells. Cancer cells show multiple phenotypes, even within the same type of cancer, there are stratifications consisting of different biological signatures, or ‘biomarkers’ to capture the CTCs and CTMs [28,34,35]. Nevertheless, the key challenge in CTCs detection is its rarity, which makes it difficult to estimate the number of single tumor cells among millions of surrounding normal peripheral blood cells. Based on their detection principle, CTCs enrichment and detection methods can be classified into cell surface marker-dependent (labeling) and marker-independent approaches (non-labeling). Most cell surface marker-dependent approaches involve positive selection, in the case of liquid biopsy of CTCs, surface protein biomarkers (Table 1) were mainly focused, followed by gene analysis or epigenetic patterns detection [36].

## 3. Application of Microfluidics Technology to CTC Isolation and Detection

With the development of fluorescent staining and microscopy technologies, more insight into the physical features of CTCs including cell deformability and cell size were studied recently. Many studies that the size of CTCs was typically larger than normal blood cells, which was utilized as a criterion for CTCs detection. Microfluidic systems offer many advantages for the separation and analysis of CTCs, especially their high level of system integration, design flexibility, material versatility and advanced degree of automation. For these reasons, a variety of microfluidic devices have been developed to separate and analyze CTCs from a liquid biopsy. [46,47].

### 3.1. Sorting Based on Size

**Figure 1:**
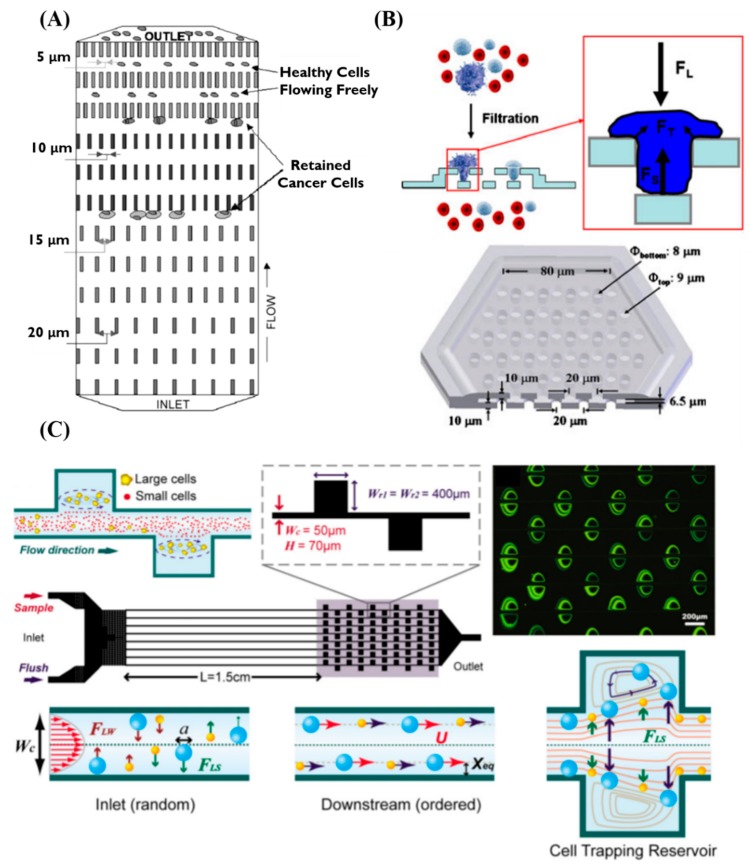
The sorting of CTCs are based on size. (**A**) The schematic device based on difference size of channel gap widths (20, 15, 10, and 5 μm) and channel depths (20, 15, 10, or 5 μm) separate cells [50]. (**B**) The schematic device of filtration process and forces on a trapped cell and a single hexagonal-shaped microfilter patch [51]. (**C**) Multiple microscale laminar vortices device and working principle were used for size-based cell isolation [52]. By adopting this technology, a High Throughput Vortex Chip (Vortex HT) [53] was developed. CTCs in whole-blood samples from advanced metastatic castration-resistant prostate cancer were isolated rapidly in this chip, which demonstrated an optimized processing speed (8 mL/min) with high purify and efficiency.

As CTCs are larger than normal blood cells, many relevant studies have used the relative size characteristics of cells to sort out CTCs [48,49]. Mohamed et al. designed a device to separate whole blood using physical property to enrich for and/or isolate rare cells from peripheral circulation. By designing arrays of different sizes, the spacing in arrays has progressively decreased (20 μm, 15 μm, 10 μm, and 5 μm), each consisting of a two-dimensional array of columns. First, using the characteristics of the microfluidic system, cells are evenly dispersed in the chip, and then the channel distance is sequentially reduced to capture smaller cells [50], as shown in Figure 1A. In another example, Zheng et al. reported a specially designed three-dimensional microfilter consisting of two layers of parylene membrane with pores and gaps to filter out small cells, the relatively larger tumor cells are left behind. Living CTCs captured on device were investigated with scanning electron microscopy, confocal microscopy, and immunofluorescent staining using model systems of cultured tumor cells spiked in blood or saline with ~86% capture efficiency in a few minutes. This unique design prevents the captured cells from being damaged by pressure and maintains their viability. In addition, it validated this 3D microfiltration concept for CTCs enrichment application [51], as shown in Figure 1B. Hur et al. developed a single-layered microfluidic device about sorting out larger cells using removal and filtering methods prevent clogging. There have been relevant studies using fluid characteristics to control the generation of eddy currents. By calculating the critical size of the cells to be captured, the size of eddy currents and initial stability can be controlled by the flow rate. This device allows larger cancer cells to be sorted out from heterogeneous cell suspensions, thereby improving overall sorting efficiency in a high-throughput manner. Using this device successfully separated larger cancer cells spiked in blood from the smaller blood cells with processing rates as high as 7.5 × 10^6^ cells/s [52,53]. (Figure 1C).

**Figure 2:**
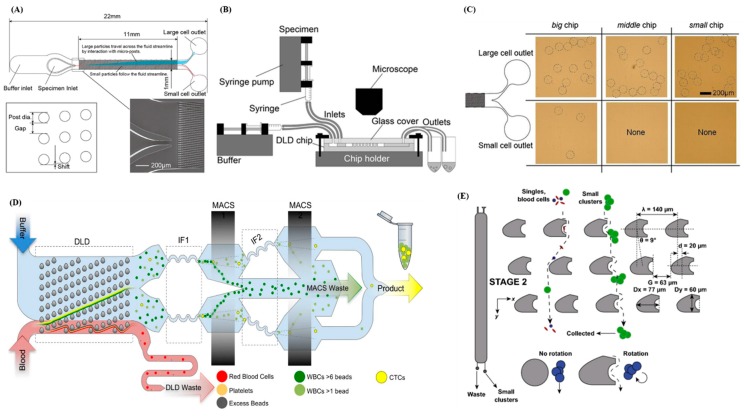
Applications of size-based DLD separation. (**A**) Schematic of DLD microfluidic device for the CTC enrichment. (**B**) Schematic of experimental setup for injecting solutions, observing the motion of samples through microscope. (**C**) Result shows the sorting of SP_2_/O (mouse myeloma cells) cultured tumor cells with “middle” and “small” configuration chips, no cell was observed in the small cell outlets, while several cells were observed in the small cell outlet with the “big” configuration chip [56]. (**D**) Schematic of five microfluidic stages includes DLD, inertial focusing stage 1 (IF1), magnetically-activated cell sorting stage 1 (MACS1), IF2 and MACS2 [57]. (**E**) The two-stage DLD arrangement based on both the size and asymmetry for CTC clusters separation [58].

Deterministic lateral displacement (DLD) is a technique in which the spacing of the microcolumn design in a chip is controlled to be smaller or larger than a critical particle diameter. Cells collide and rebound in the array differently owing to their different volumes and masses. Larger particles with higher masses have smaller rebound angles, whereas smaller particles with lower masses have larger rebound angles. After successive collisions, clusters of particles of different sizes can be sorted out [54,55]. Okano et al. attempted to use DLD microfluidic device for enrichment of CTCs in tumor-bearing mouse blood. This method used blood samples were prepared from tumor-bearing mice. Tumors were induced by injecting cells with green fluorescent protein (GFP) expression to living mouse. It has significant result in the enrichment of tumor cells to 0.05% from the blood. CTCs were negligibly detected among three million blood cells, and no obvious leakage of CTCs was observed (Figure 2A–C) [56]. In another work, Fachin et al. presented an automated monolithic chip with 128 multiplexed DLD devices containing ~1.5 million microfabricated features (12 µm–50 µm) used to exhaust RBCs, platelets and WBCs. It quantified the size and EpCAM expression of over 2500 CTCs from 38 patient samples obtained from breast, prostate, lung cancers, and melanoma and found that neither CTC size nor EpCAM expression can maximize isolation efficiency as many CTCs found were small and expressed lower levels of EpCAM (Figure 2D) [57]. Au et al. presented a two-stage continuous microfluidic chip that separates and recovers viable CTC clusters from blood. This method uses DLD to sort clusters by using two geometric attributes (size and asymmetry). Using this integrated two-stage device, cultured breast cancer CTC clusters containing 2–100 + cells were recovered from whole blood with minimal cluster dissociation, 99% large cluster recovery, cell viability exceeding 87% and greater than five-log depletion of RBCs (Figure 2E) [58]. Related studies have indicated that the principle of DLD can be applied with arrays of different shapes to improve the efficiency of CTC isolation.

### 3.2. Immunoaffinity-Based CTCs Isolation and Detection

CTCs can be captured from a variety of cells by taking advantage of the interaction between specific antigens and antibodies [59]. These antigens are expressed in most CTCs and do not appear in other cells such as WBCs and RBCs. With a microfluidic chip as the substrate, its superior surface area is modified with a selective antibody. When tumor cells flow with the fluid over the antibody-modified surface, the target cells are captured by the interaction between the antibodies and the antigen, and the remaining blood cells continue flowing with the fluid to the outlet [60].

**Figure 3:**
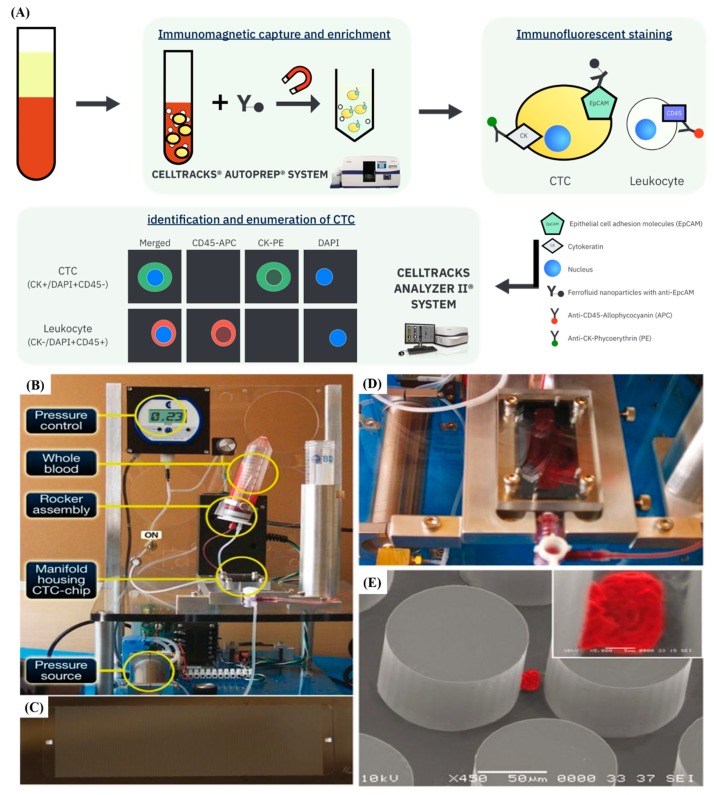
CTCs can be captured from a variety of cells by taking advantage of the interaction between specific biomarkers. (**A**) 7.5 mL of blood sample is centrifuged to separate solid blood components from plasma, and then placed in the CELLTRACKS^®^ AUTOPREP^®^ System to magnetically separate CTCs from other blood cells by using ferrofluid nanoparticles with EpCAM antibodies. The separated EpCAM^+^ CTCs are then stained with CK mAb and contaminating leukocytes are identified with CD45. After a DNA stain (DAPI), stained CTCs are put in a magnet cartridge and placed onto the CELLTRACKS ANALYZER II^®^ System for scanning. Tumor cells that are positive for CK and DAPI would be displays by the system. (**B**) The workstation setup with a microfluidic device for isolating CTCs from the blood of patients with common epithelial tumors. (**C**) The CTC-chip with microcolumns which are made chemically functional with EpCAM antibodies was etched in silicon. (**D**) The microfluidic device with a manifold to enclosed the chip and a pneumatic pump to build the whole blood flow through the capture module. (**E**) The inset shows a high magnification view of the scanning electron microscope image of a captured NCI-H1650 lung cancer cell spiked into blood (pseudo-coloured red). [64].

In recent years, this has been combined with other technologies such as microarray designs and magnetic devices. The CellSearch system combines antibodies (EpCAM) with magnetic beads functionalized through surface modification. Upon binding with target cells, an external magnetic field can be applied to capture the cells. The captured cells are immunocytochemically stained with DAPI, cytokeratin 8/18/19, and leukocytes are excluded by using the pan-leukocyte marker CD45 [61] (Figure 3A). This method has been proven to be highly specific, and its performance remains stable at CTC counts as low as 1 CTC/7.5 mL of blood while ensuring minimal inter-reader variability [62]. Estes et al. reported antibody-coated magnetic beads to capture target cells in stages. The device consisted of two separate chambers, and the different chambers were combined with different antibody-coated magnetic beads. Taking prostate cancer as an example, the specific markers used were PSMA and CD10. When flowing through the first chamber, cells in the cell suspension expressing CD10 bind to the magnetic beads, and when flowing through the second chamber, cancer cells expressing PSMA were captured, thereby allowing different subtypes of cell populations to be respectively captured using this two-stage design [63].

Cell-affinity chromatography is a method for capturing cancer cells from a heterogeneous cell suspension. The substrate surface is functionalized by modifying the substrate with a target cell-specific high-affinity ligand, thereby allowing target cells to selectively adhere to the microfluidic chip [28]. In Nagrath et al.’s studies, such CTC chips were designed with a series of about 78,000 independent microcolumns to provide a large surface area for coating with EpCAM antibody. This device can be used repeatedly to isolate adherent CTCs from the blood with a capture rate of about 50% (Figure 3B–E) [64].

**Figure 4:**
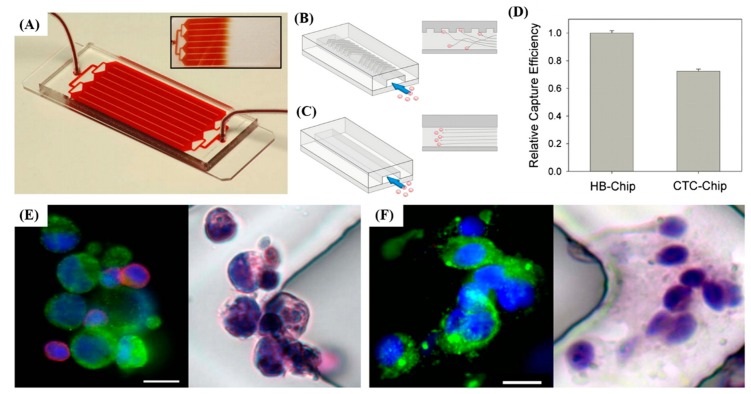
A platform for CTC isolation. (**A**) The schematic is consisted of a microfluidic array of channels with a single inlet and outlet showed the uniform blood flow through the HB-Chip. (**B**) Schematic of the cell-surface interactions in the HB-Chip. (**C**) Schematic of a traditional flat-walled microfluidic device. (**D**) The HB-Chip improved capture efficiency in comparison with the CTC-Chip. (26.3% improvement, p = 0.0001) (**E**) Photographs showing a CTC cluster isolated from a metastatic prostate cancer patient on the HB-Chip; immunofluoresence staining (DNA (blue), prostate-specific membrane antigen (green), and CD45 (red)) and subsequent immunohistochemical staining (Hematoxylin and Eosin staining, H & E staining) are shown. (**F**) A CTC cluster isolated from a metastatic lung cancer patient on the HB-Chip; immunofluoresence staining (DNA (blue), cytokeratins 7/8 (green), and CD45 (red)) and subsequent immunohistochemical staining (H & E staining) are shown. All scale bars represent 10 μm. [66].

In addition, Myung et al. aimed to use a nanotechnology-based assay (CapioCyte) functionalized with aEpCAM, aHER-2 and aEGFR to detect the effects of multivalent binding and biomimetic cell rolling on the sensitivity and specificity of CTC capture [65]. CapioCyte used multivalent binding via poly(amidoamine) dendrimers further improved capture sensitivity and showed that cell rolling effect can improve CTC capture specificity (% of captured cells that are CK+/CD45−/DAPI+) up to 38%. Stott et al. designed a second-generation CTCs chip known as a herringbone chip (HB Chip) which is designed to generate micro-eddy currents to increase the interaction between cells and the antibody-coated surface. CTCs were detected in 14 of 15 (93%) patients with metastatic disease (median = 63 CTCs/mL, mean = 386 ± 238 CTCs/mL), and the tumor-specific TMPRSS2-ERG translocation was readily identified following RNA isolation and RT-PCR analysis. As fluids experience laminar flow in traditional chips, cells follow a single streamlined flow, and a very small number of cells will cross the streamline; therefore, the number of cells that interact with the surface is limited. In contrast, the herringbone design can increase the overall capture and adhesion rate (Figure 4A–F) [66]. The application of surface biomarkers and microfluidic technology to isolate CTCs provides an opportunity for enhanced capture efficiency, kept these rare cells viability, and further explored the potential mechanism in different cancer types and progression (Table 2).

### 3.3. Dielectrophoresis (DEP)

**Figure 5:**
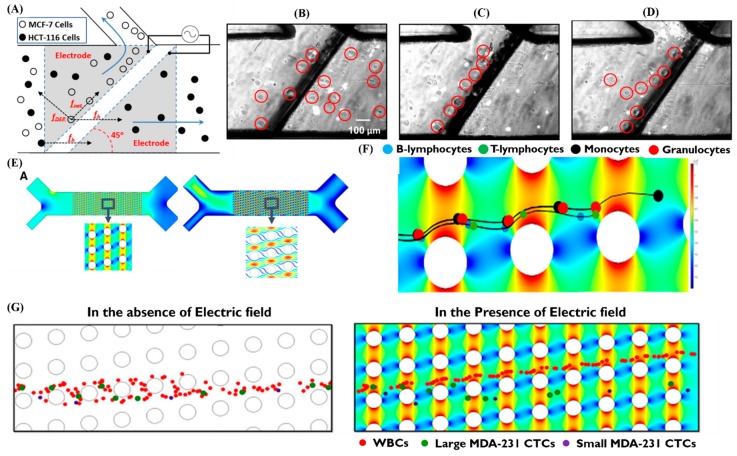
Application of DEP for separating CTCs. (**A**) Schematic of a dielectrophoretic lab-on-a-chip device was rely on a DEP and a hydrodynamic force. Arrows show flow through the main channel (80% infusion rate) and through the side channel (20% infusion rate), respectively. (B-D) The circles are used to mark MCF-7. (**B**) With no separation of the MCF-7 cells from the HCT-116 moved through the main channel without AC activation of the electrodes. (**C**) The MCF-7 cells are aligned parallel to the electrode (dark region) and are being deflected alone the side channel but the HCT-116 path is unchanged when the power source was turned on at 3.2 MHz. (**D**) Shows the system is really sensitive. Small changes in AC frequency is 2.0 MHz and can significantly change the results between the MCF-7 and HCT-116. [70] (**E**) Shows the electric field norm (left) and velocity field (right) of the DLD-DEP device. (**F**) Granulocytes, T-lymphocytes, B-lymphocytes, and monocytes were presented in the system and their paths were captured in the presence of applied electric field. (**G**) WBCs and CTCs’ trajectory in the absence of electric field (left), in the presence of electric field (right) by applying electric, CTCs can be separated from WBCs by changing the trajectory of WBCs. The design is capable of CTC separation from WBCs with similar size [72].

Dielectrophoresis (DEP) uses different degrees of polarization produced between cells to apply a non-uniform alternating electric field. After the cells are subjected to the electric field, the cell charges interact with the electric field and are separated by moving toward electric fields of different intensities. The magnitude of the dielectrophoretic force is determined by the cell membrane, cytoplasm, and even cell size, so DEP is often used to isolate cancer cells in microfluidic chips [68,69]. Alshareef et al. used a dielectrophoretic lab-on-a-chip device for separating CTC. This method requires to make a control of frequency effect (3.2 MHz), voltage effect (9 Voltage, Vpp) and flow rate effect (0.1 μl/min) to realize enrichment efficiencies as high as 93% for distinguishing and separating MCF-7 human breast cancer cells from HCT-116 colorectal cancer cells. The platform could not only show a potentially label free, but also real-time and cost-effective for diagnosis of CTC (Figure 5A–D) [70].

In a study using dielectrophoretic properties to adsorb cancer cells from a cell suspension onto a microelectrode array, it was found that cancer cells were significantly different from most blood cells, most of which could be removed, and in the end, the cancer cells can be collected by removing the applied electric field [71]. Aghaamoo et al. designed a deterministic DEP method that combines the concept of deterministic lateral displacement (DLD) and insulator-based dielectrophoresis (iDEP) techniques that depend on physical properties such as size and dielectric for continuous separation of CTCs from peripheral blood cells. This study try to use numerical modeling, different design of combined DLD and DEP such as the required applied voltages, velocities, and geometrical parameters of DLD arrays provides continuous sorting of CTCs from WBCs even with similar size and has the future potential for high throughput and efficiency (Figure 5E–G) [72].

The methods introduced above are often used in current studies of microfluidic cancer detection systems. Despite advances in various novel technologies, systems capable of simultaneously achieving high throughput, high target cell viability, and high collection purity are still scarce. Although capture methods based on biological surface modification have unique chip designs, such as the herringbone design, 3D nanomicrostructure, and microarray column designs, that can enhance the contact area between cells and antibodies to achieve high specificity and high purity of capture, they are limited primarily owing to their low flow rates and low flow throughputs; furthermore, sample testing is very time-consuming as a result. Although physical methods based on filtering have high flow rates, high throughputs, and higher capture purity compared to capture methods using biological surface markers, owing to physical extrusion, the cell viability of the captured cells is low. This causes cell deformation and damage and renders them unable to be subsequently cultured and studied. DEP technology separates cells by their size differences and dielectric properties and has higher separation purity compared to filtration techniques, but the dielectric difference between the target cells and other cells is limited after implementation; moreover, the sorting efficiency differs greatly from expectations. To overcome these limitations, a microfluidic system that integrates the advantages of various physical and biological techniques must be developed to achieve high purity, high recovery rate, and high cancer cell capture rate.

## 4. Application of Nanomaterials in Microfluidic-Based Systems for Enhanced CTCs Isolation and Detection

Many studies on nanomaterials have been widely conducted in different scientific fields. Nanomaterials have unique physicochemical properties and biocompatibility that are significantly different from those of monoatomic or generic bulk structures. This difference is caused by different spatial arrangements, shapes, phase changes, electronic structures, and other factors. After surface modification, nanomaterials can be combined with a variety of reactive functional groups to further interact with antibodies, peptides, and protein receptors to construct multifunctional hybrid nanomaterials for biologically targeted cancer therapies, multiple detectors, biosensors, and so on [73]. Among them, the development of nanoparticles for medical diagnostics has been the most extensive [74]. Owing to the size advantage of nanoparticles, they can be used to detect the release of high-concentration biomarkers and indirectly amplify the signal to improve the detection rate, even in the early stages of cancer [75]. In addition, they can also be applied in the early diagnosis of brain cancer [76]. By combining nanoparticle and nuclear magnetic resonance (NMR) technologies, specially modified magnetic nanoparticles adhered to particles in the blood known as microvesicles that originate from brain cancer. They were detected by NMR scanning, thereby achieving early diagnosis. Some studies have used nanomaterials to detect viruses, bacteria, or chemical mixtures in combination with surface-enhanced Raman spectroscopy [77]. Each compound has its own unique frequency and Raman shift, and silver nanorods are used to significantly amplify the signal, thereby distinguishing various viruses, bacteria, and biomarkers.

**Figure 6:**
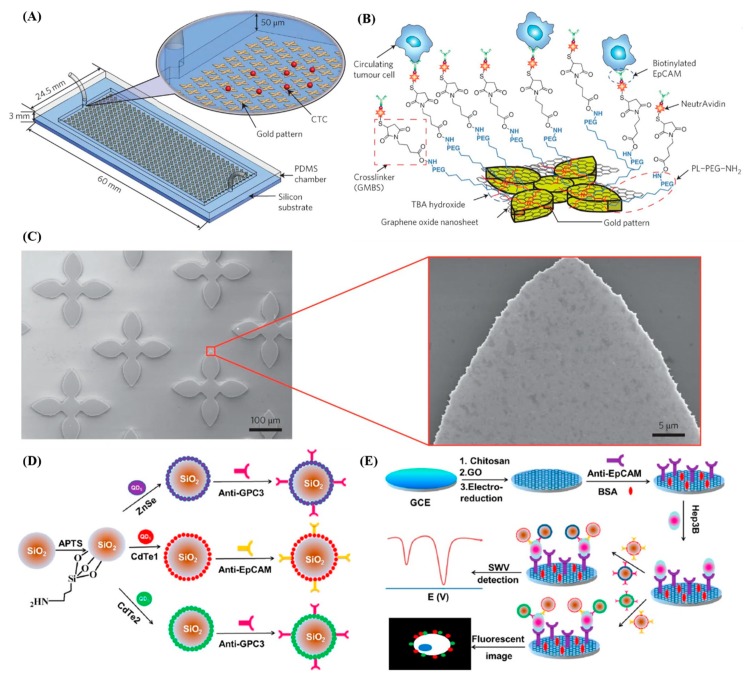
Application of nanomaterials to CTCs diagnostics. (**A**) Schematic of the GO chip. The silicon substrate has 58,957 flower-shaped gold patterns with dimensions of 100 µm × 100 µm. (**B**) Schematic showing the chemical conjugation between functionalized GO nanosheets and the EpCAM antibodies. GO nanosheets are adsorbed onto the gold pattern. The N-γ-maleimidobutyryloxy succinimide ester (GMBS) crosslinker binds to PL–PEG–NH2 on the GO nanosheets. The NeutrAvidin is connected to the GMBS and biotinylated EpCAM. (C) Left: Scanning electron microscope (SEM) images shows the gold patterns. Right: magnified SEM image shows adsorbed GO nanosheets on gold patterns [67]. (**D**) Schematic of the preparation of Si/Zn/anti-GPC3, Si/Cd1/anti-EpCAM, and Si/Cd2/anti-GPC. (**E**) Ultrasensitive graphene-enhanced fluorescent and electrochemical CTC detection procedures [84].

In addition, graphene oxide (GO) is also a promising nanomaterial with applications in biosensors because of its ease of surface modification, good biocompatibility, hydrophilicity, and sheet-like structure that provides a large surface area [72,78,79,80,81]. Its surface also contains a series of oxygen-containing functional groups, including carboxy, epoxy, and hydroxy functional groups, which bind easily with biological targets such as antibodies and peptides to form biosensors due to the abundant oxygen functional groups. In the past few years, various types of functional GO nanomaterials have been used in the design of various structures as CTC diagnostic platforms [82]. Yoon et al. have utilized GO nanosheets adsorbed on a patterned gold surface as a substrate with antibodies chemically linked in order to capture rare CTCs in the blood. CTCs were captured with high sensitivity at a low concentration of target cells (73 ± 32.4% at 3-5 cells per ml blood) [67]. (Figure 6A–C). The same group also use this GO chip-based CTC assay to isolate and characterize CTCs from 47 metastatic breast cancer patients. The CTCs are captured through multimarker analysis using IF staining and RT-qPCR indicated involved in metastasis including EMT and apoptotic-resistant mechanisms, which correspond with previous findings. This current study is the first step toward translating GO platform into clinical settings, facilitating understanding of metastasis and helping to identify pathways relevant to potential therapeutic targets for personalized therapy [83].

Studies have shown that this GO chip can achieve isolation and capture of CTCs. Similarly, Wu et al. presented electrochemical and fluorescent signal transduction was used to design a dual-signal amplification sensor chip using graphene as a substrate [84]. The graphene-modified surface accelerates electron transfer and enhances signal detection, and CdTe- and ZnSe-coated silica nanoparticles have uniform size distribution, good stability coated with quantum dots (QD) were used as tracking tags to label anti-EpCAM and anti-GPC3, thereby using two methods to improve detection sensitivity (Figure 6D). Amino groups on the chitosan membrane enabled covalent binding of the capture antibodies (Figure 6E). Moreover, the binding between the antibody and CTC is non-covalent, but offers the ability to detach the bound CTCs. It has significant high sensitivity, specificity to detect low-abundance tumor cells with stability, reproducibility, and accuracy for clinical diagnostics.

Because nanomaterials are widely used in biosensors and diagnostic platforms, surface modification is essential to improving the sensitivity of nanomaterials [85]. The efficiency of use is determined by the chemical properties of the material surface, the conjugated biological or chemical molecules used for composition, the functional groups present on the biomolecules, and the ease of immobilization on the surface of modified materials. Commonly modified surface substrates include silicon, glass slides, carbon, polystyrene, gold, and others [86]. General modification methods include physical adsorption methods and using π-π interactions between hydrophobic amino acids in the protein or enzyme, and the material allows it to adsorb to and be immobilized on the substrate surface. Through non-covalent interactions, fluorescent molecules can be immobilized to the surface. In order to avoid non-specific binding, covalent modification techniques are still available, which are divided into direct and indirect methods, including carboxyl [87], amine [88], and epoxy [89] modifications, providing linkage for subsequent bonding through reagent activation or conversion into multiple functional groups. More common modification methods include biotin-avidin coupling and click chemistry. Biotin-avidin coupling is the most commonly used coupling method in the laboratory. Avidin has multiple biotin binding sites and can bind to remaining biotin ligands through their free binding sites. In other words, if biotin is on the surface, nearly all biomolecules can be immobilized onto the target using avidin protein labeling. The interaction is strong despite being a non-covalent interaction, so it is nearly unaffected by pH, temperature, or organic solvents [90]. They are combined with each other through the sandwich method, and the extent of their application is primarily as a substrate for biosensors, affinity targeting studies, and biomedical diagnostics [91]. Generally, the interaction is performed using a commercial kit, and biotin is added to the amino groups on the surface of the peptide. As a result, there is no guarantee that there will be only one biotin molecule on each ligand, and biotin may also bind to the remaining sites, resulting in decreased specificity and selectivity [92].

Click chemistry is a new and emerging technology in the field of chemical synthesis that was originally applied in the development of pharmaceutical synthesis. The concept originates from the rapid chemical bonding between carbon and heteroatoms (atoms other than carbon and hydrogen). This reaction can be used to covalently link two structures with very different chemical properties, such as compounds, biomolecules, and even reagents, thereby forming structures completely different from the original molecule [93]. It is widely used in radiochemistry, proteins, nucleic acids, and clinical medicine [94]. Click chemistry has many advantages, including bioorthogonality, meaning that the functional groups of its reactants and its products do not interact with functional biomolecules [95]. This reaction is irreversible and produces products with high specificity and high stability. In addition, the reaction can be prepared at room temperature, has low toxicity and high biocompatibility, and is not affected by excess byproducts.

**Figure 7:**
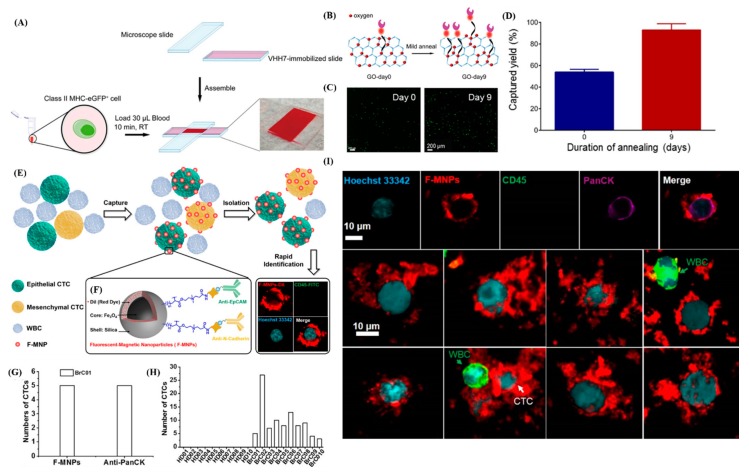
Application of material surface modification methods to diagnostics. (**A**) Schematic of the cell capture device made from glass slides, the assay conditions, and a digital photograph of the constructed and loaded capture chamber. (**B**) Schematic showing enhanced functionalization of GO nanosheets because of oxygen clustering on the graphene basal plane. GO-day 0 represents as-synthesized GO sheets with no annealing treatment, while GO-day 9 represents GO substrates annealed for 9 days at 80 °C to induce oxygen clustering. (**C**) Quantification of captured class II MHC-positive eGFP+ cells from murine whole blood samples comparing Day 0 and Day 9 substrates. (**D**) Cell capture efficiency (92 ± 7%) of annealed GO structure (Day 9) compared to that of as-synthesized structure (Day 0, 54 ± 3%) [100]. (**E**) Schematic for (E) the isolation and rapid identification of heterogeneous CTCs using the F-MNPs and (**F**) the construction as well as chemistry modification of F-MNPs. (**G**) Comparison of identification performance of immunostaining and F-MNPs for the patient sample. (**H**) CTC count of healthy donors and breast cancer patients identified by dual-antibody-modified F-MNPs. (**I**) Fluorescent images of a CTC identified by immunostaining of anti-PanCK-647 as well as F-MNPs and fluorescent images of eight CTCs (Hoechst 33342+, F-MNPs+, CD45−) and WBCs (Hoechst 33342+, CD45+) from the patient sample identified by F-MNPs with immunostaining of anti-CD45-FITC and Hoechst 33342 [103].

Among the many click chemistry reactions, the Hüisgen 1, 3-dipolar cycloaddition reaction, which combines two unsaturated reactants, is the most popular [94]. Initially, a high-temperature and high-pressure environment is required for the reaction to proceed without a catalyst [96], until it was found that using copper Cu(I) as a catalyst could increase the reaction rate at room temperature significantly, but the high toxicity of copper limits its application *in vivo*. Subsequently, studies have been conducted using click chemistry without the need for metal catalysts, including copper(I)-catalyzed azide-alkyne cycloaddition, also known as the CuAAC method [97], in which ppm levels of catalyst are still present in the product after purification, as well as the strain-promoted azide-alkyne [3 + 2] cycloaddition reaction, also known as the SPAAC method. An example is the reaction between dibenzocyclooctyl (DBCO) and azide [98]. This new technique for covalent linkage does not require a metal substance as a catalyst. In some studies, DBCO was linked to one end of DNA [99] and then covalently bonded to azide for biomolecule calibration. This linkage was found to have a high degree of mechanical stability and also demonstrates that click reactions can occur without interfering with natural biochemical processes that occur in organisms *in vivo*, which is tremendously beneficial for future diagnostic applications. Previously, a research group had used GO as a substrate in combination with single-domain antibody, protein modification, and click chemistry technologies so that the antibody can be immobilized unidirectional on the GO substrate, thereby establishing a functional device that can rapidly capture a small number of specific cells from the blood [100]. Other related studies have demonstrated that when antibodies are immobilized to a substrate by click chemistry, target specificity and sensitivity are greatly enhanced in addition to effectively maintaining the activity of the biological probe [101,102] (Figure 7A–D).

This confirms the advantages of combining nanomaterials with interfaces modified using click chemistry for liquid biopsy. Wang et al. developed a method is to isolate and identify heterogeneous CTCs with high efficiency from patient blood samples using the fluorescent-magnetic nanoparticles (F-MNPs). The F-MNPs are fabricated to conjugate a dual-antibody interface targeting EpCAM and N-cadherin to capture epithelial CTCs and mesenchymal CTCs from whole blood samples. The F-MNPs were validated in CTC isolation and CTC identification in the blood samples of cancer patients [103] (Figure 7 E–I).

## 5. Conclusions and Perspectives

In cancer, early detection of tumors is the best prevention and treatment for patients who are still asymptomatic. However, early detection of tumors via either CTCs or CTMs has their respective challenges. Isolated cancer cells in the blood are very rare, traditional cell separation methods require many cumbersome steps such as RBC lysis and centrifugation, resulting in the loss of the original in vivo characteristics of the cells thus isolated. These “laboratory-only” methods eventually lead to a bottleneck in the diagnosis of cancer cells and causes the clinical significance of CTC/CTM to decrease continuously. In addition, deeper investigation of the cells isolated as CTCs and CTMs is rare in current studies even though cell separation techniques are continuously evolving. Therefore, the integration of microchannels, nanomaterials, and surface modification technologies will be an important future research direction. By combining the advantages of interdisciplinary methods, the bottlenecks in traditional cancer cell sorting methods can be resolved. In addition to effectively sorting out cancer cells from the blood, maintaining the type and characteristics of the cells and further elucidating the role of CTCs and CTMs in cancer metastasis is the only way to benefit clinical diagnostic medicine by achieving the original intention of using cells from liquid biopsy for diagnosis.

## Figures and Tables

**Table 1:** Clinical studies that have explored phenotype of CTCs.

Phenotype	Marker	Reference
**Epithelial-like**	EpCAM	[37]
	Cytokeratin	[38]
	E-cadherin	[39]
	Zonula occludens	[40]
	Epithelial splicing regulator1	[41]
**Mesenchymal-like**	Vimentin	[23]
	N-cadherin	[24]
	Twist1	[42,43]
	ZEB1	[44]
	Plastin-3	[45]

**Table 2:** Immunoaffinity-based technologies for CTCs isolation and detection.

Strategies	Technology	Selection Criteria	Biomarkers used for CTCs Characterization after Processing	Throughput	Performance in Spiking Experiment		Performance in Clinical Samples		Reference
					Sample	Capture Efficiency (Captured CTCs/Total CTCs in Samples)	Sample	Sensitivity (Captured CTCs/Total Volume of Sample or Total)	
**Microfluidics - DLD**	**monolithic CTC-iChip**	**Size cutoff of 3.8 µm and CD45^-^/CD16^-^/CD66^-^**	**DRAQ5^+^/EpCAM^+^ (Prostate-Lung-Breast)/ CK/Her2 (Breast)/ CD146/NG2^+^ (Melanoma)**	**~15 million cells/second**	11 different cell lines (SkMel28, H1650, H1975, H3122, LNCAP, PC3, PC3-9, VCAP, MB231, MCF-7, SkBR), spiked in 1X PBS with 1% F68 (average concentration: 425 cells/mL)	99.5%	Whole blood from cancer patients	1~63 CTCs/ mL	[57]
cancer-specific antibodies-coated magnetic beads	CellSearch	EpCAM^+^	CD45^-^/CK^+^/DAPI^+^	-	1000 or 4000 human tumor cells spiked in 7.5 mL whole blood, diluted with 6.5 mL of dilution buffer (Veridex)	-	-	-	[61]
cancer-specific antibodies-coated magnetic beads	On-chip magnetic bead-based cell separator	PSMA^+^/CD10^−^	-	2-200 µL/ min	1 million cells in 100 µL PBS buffer	60 ± 10%	-	-	[63]
Microfluidics + cancer-specific antibodies-coated microposts	CTC-chip	EpCAM^+^	CK^+^/CD45^-^/DAPI^+^	1~2 mL/ h	Whole blood spiked with cancer cells (concentration ranging from 50 to 50,000 tumor cells per mL)	>60%	Whole blood from cancer patients	1 target cell/ 2.7 mL	[64]
cancer-specific antibodies immobilized-surfaces functionalized with dendrimers	CapioCyte	E-selectin^-^/EpCAM^+^/HER-2^+^/EGFR^+^	CK^+^/ CD45^-^/ DAPI^+^	25 µL/ min	-	-	Whole blood from cancer patients	19~849 CTCs/ mL	[65]
Cancer-specific antibodies-coated microposts	HB Chip	EpCAM+	CK^+^/CD45^-^/PSA^+^/CEPX^+^/AR^+^/DAPI^+^	1.5~2.5 mL/h	PC3 cells spiked into whole blood	91.8% ± 5.2%	Whole blood from metastatic prostate cancer patients at various stages of treatment	12~3167 CTCs/mL	[66]
Nanomaterial-interfaced patterned gold surface	GO-interfaced patterned gold surface	EpCAM+	CK^+^/CD45^-^	1~3 mL/ h	Fluorescent tracker dyed MCF-7 and PC-3 spiked into buffer	82.3%	Whole blood samples from patients with metastatic breast cancer, early stage lung cancer and metastatic pancreatic cancer	1~23 CTCs/ mL	[67]
